# The DEAD-box RNA Helicase DDX6 is Required for Efficient Encapsidation of a Retroviral Genome

**DOI:** 10.1371/journal.ppat.1002303

**Published:** 2011-10-13

**Authors:** Shuyuarn F. Yu, Phillip Lujan, Dana L. Jackson, Michael Emerman, Maxine L. Linial

**Affiliations:** 1 Division of Basic Sciences, Fred Hutchinson Cancer Research Center, Seattle, Washington, United States of America; 2 Division of Human Biology, Fred Hutchinson Cancer Research Center, Seattle, Washington, United States of America; Duke University Medical Center, United States of America

## Abstract

Viruses have to encapsidate their own genomes during the assembly process. For most RNA viruses, there are sequences within the viral RNA and virion proteins needed for high efficiency of genome encapsidation. However, the roles of host proteins in this process are not understood. Here we find that the cellular DEAD-box RNA helicase DDX6 is required for efficient genome packaging of foamy virus, a spumaretrovirus. After infection, a significant amount of DDX6, normally concentrated in P bodies and stress granules, re-localizes to the pericentriolar site where viral RNAs and Gag capsid proteins are concentrated and capsids are assembled. Knockdown of DDX6 by siRNA leads to a decreased level of viral nucleic acids in extracellular particles, although viral protein expression, capsid assembly and release, and accumulation of viral RNA and Gag protein at the assembly site are little affected. DDX6 does not interact stably with Gag proteins nor is it incorporated into particles. However, we find that the ATPase/helicase motif of DDX6 is essential for viral replication. This suggests that the ATP hydrolysis and/or the RNA unwinding activities of DDX6 function in moderating the viral RNA conformation and/or viral RNA-Gag ribonucleoprotein complex in a transient manner to facilitate incorporation of the viral RNA into particles. These results reveal a unique role for a highly conserved cellular protein of RNA metabolism in specifically re-locating to the site of viral assembly for its function as a catalyst in retroviral RNA packaging.

## Author Summary

Foamy viruses are complex retroviruses that infect non-human primates, cats, cows, and horses. Humans are not natural hosts but can acquire primate foamy viruses as zoonotic infections. During foamy virus assembly process, viral RNAs and Gag capsid proteins are targeted to a discrete intra-cytoplasmic site where viral particles are assembled. One key step in this process is to effectively incorporate the virus genome into particles. For retroviruses, encapsidation of viral genomic RNA is known to initiate when specific packaging sequences within the viral RNA are recognized by the nucleocapsid domain of the Gag polypeptide. However, the contribution of host factors to the assembly process is largely unknown. In this study, we find that after foamy virus infection some of the cellular DEAD-box RNA helicase DDX6 specifically re-localizes to the viral assembly site, and is needed for efficient packaging of viral RNA into particles. Our data suggest that the ATP hydrolysis and RNA unwinding activities of DDX6 function in remodeling the structure of viral RNA and/or RNA-Gag ribonucleoprotein to facilitate its incorporation into particles. Our work provides the first report of an evolutionarily conserved host protein involved in the assembly of retrovirus genomes into particles.

## Introduction

Foamy viruses, the only genus in the retrovirus subfamily *Spumaretrovirinae*, are complex retroviruses that infect all non-human primates, cats, cows, and horses. Humans are not natural hosts but can acquire primate foamy viruses as zoonotic infections [1]. The genome of foamy virus encodes three structural proteins, Gag, Pol, and Env, as well as other regulatory proteins. Gag protein, as in all other retroviruses, is the major structural protein for formation of viral capsids. However, the Gag precursor protein of foamy virus is not cleaved into matrix, capsid, and nucleocapsid subunits which are characteristic of orthoretroviral Gag in mature virions. Instead, cleavage of foamy virus Gag is rather inefficient during maturation, leading to a cleavage product of p68 polypeptide by removing 3 kD from the C-terminal end of p71 Gag precursor protein [2]. The assembly pathway of foamy virus is similar to that of betaretroviruses such as Mason-Pfizer monkey virus [3]. The newly synthesized Gag capsid proteins of foamy virus are targeted to an intra-cytoplasmic site near the centrioles, also known as microtubule organizing center (MTOC), where they are assembled into capsids [4]. Although lentiviruses such as human immunodeficiency virus (HIV) assemble at the plasma membrane, the initial interaction of Gag with viral RNA is thought to occur at the pericentriolar site [5]. This intracellular region near the MTOC could serve as a scaffold for recruitment of viral elements and cellular factors for viral replication and assembly. One key step during the assembly process is to incorporate the virus genome into particles. For retroviruses, packaging is usually initiated when a *cis*-acting packaging signal in the viral RNA is recognized by the nucleocapsid domain of Gag. While packaging sequences in the RNA and protein motifs in the nucleocapsid protein are well studied, the contribution of host factors to the process of viral RNA packaging is largely unknown.

In eukaryotic cells, translationally repressed mRNAs are often sequestered in discrete cytoplasmic RNA granules called P bodies, where they are either degraded or stored for later translation. If mRNAs are stalled in a translation preinitiation complex (in response to stresses like heat shock, oxidative stress, UV irradiation, or viral infection), they are packaged into another form of granules named stress granules. Components of P bodies and stress granules are closely associated with RNA metabolism. P bodies include components of the 5′-3′ mRNA degradation pathway such as Dcp1, Dcp2, Ago1, Ago2, MOV10, and DDX6 (reviewed in [6]). Stress granules contain many of the early translation initiation factors including the poly(A)-binding protein PABPC1. When cells are subjected to stresses, some P body proteins like Ago1, Ago2, MOV10, and DDX6 can be redistributed to stress granules (reviewed in [7]). Proteins associated with P bodies and stress granules also play important roles in the life cycles of viruses as an antiviral defense or a requirement for viral replication (reviewed in [8]). For example, overexpression of MOV10, a putative RNA helicase, can inhibit replication of HIV and other retroviruses at multiple stages including reverse transcription [9]–[11]. In contrast, several P body proteins including Dhh1p, the yeast homologue of DDX6, are required for retrotransposition and virus-like particle assembly in yeast retrotransposons Ty1 and Ty3 [12]–[15].

DDX6, also known as RCK/p54, is an abundant protein found bound to non-translating mRNA in the cytoplasm, although it is often concentrated in both P bodies and stress granules (reviewed in [16]). DDX6 belongs to the DEAD-box RNA helicase family, named after a highly conserved Asp-Glu-Ala-Asp (D-E-A-D) amino acid motif. These proteins contain nine conserved motifs that together are responsible for the RNA-dependent ATPase and ATP-dependent RNA helicase activities. The DEAD-box helicases are involved in various RNA-related biological processes as a result of their ability to mediate conformational changes of their RNA substrates through ATP hydrolysis, and to unwind double-stranded RNA duplexes via the helicase activity (reviewed in [17]). Members of the helicase family are known to play different roles in the replication of viruses. For example, DDX6 and another helicase DDX3 are both required for efficient replication of hepatitis C virus [18]–[20]. In HIV, DDX6 is implicated in negatively regulating viral replication [21], [22], whereas DDX3 can enhance Rev-dependent nuclear export of unspliced viral transcripts by interacting with Rev [23].

Our previous work has shown that capsid assembly of prototype foamy virus (PFV) occurs mainly in the cytoplasm near the MTOC where Gag capsid protein accumulates [4]. In this study, we have explored the roles of cellular proteins within P bodies and stress granules in viral replication and assembly. We demonstrate that the DEAD-box RNA helicase DDX6 re-localizes to the site of foamy virus assembly where viral RNA and Gag proteins are concentrated, and is required for encapsidation of virus genome during the assembly process.

## Results

### Effects of P body and stress granule proteins on viral infectivity

**Figure 1:**
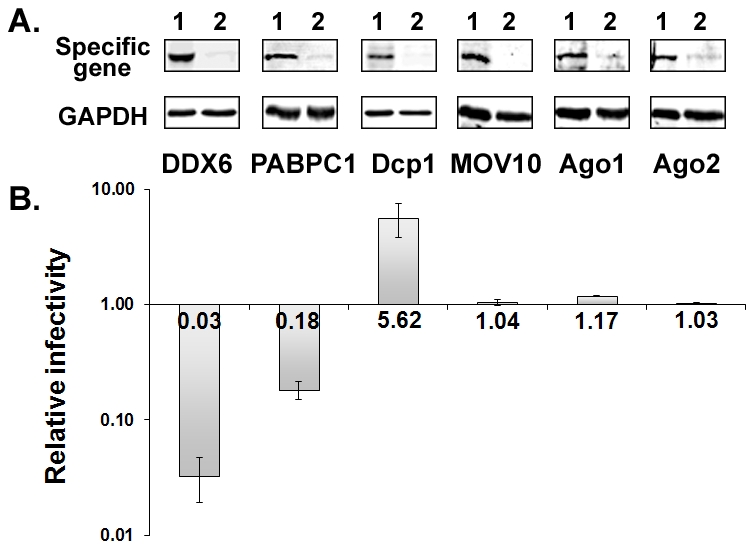
Effects of gene-specific siRNA knockdown on viral infectivity. (A) HT1080 cells were transfected twice with 120 nM control siRNA (Lanes 1) or specific siRNA (Lanes 2). 1^st^ siRNA transfection was performed at time 0 and 2^nd^ siRNA transfection at 24 h. At 24 h after the 2^nd^ transfection, half of the cells were infected with virus and the other half were analyzed by immunoblot for each specific protein (top panels) and GAPDH (bottom panels). (B) Culture supernatant was harvested at 48 h after infection and used to infect FAB cells to measure infectious titers. Relative infectivity of viruses derived from cells transfected with specific siRNA compared to those with control siRNA. Means and standard errors from at least four separate experiments are shown.

Six P body and stress granule proteins were individually silenced using gene-specific siRNA to examine whether or not they were required for replication of prototype foamy virus (PFV). PFV was originally isolated from a human tumor cell line, but is now known to be a chimpanzee foamy virus that was zoonotically transmitted to the human from whom it was cultured [24]. HT1080 cells were transfected twice with 120 nM control or specific siRNA. At 24 h after the 2^nd^ siRNA transfection, cells were infected with PFV for 48 hrs, and culture supernatants were analyzed by the FAB assay to measure infectious titers [25]. Cytotoxic effect was found after transfection with the siRNA; however, it was mainly caused by the transfection procedure, especially the amounts of siRNA and transfection reagents, since cells transfected with the control siRNA exhibited a similar level of effects as specific siRNAs. Although cell morphology changed slightly by DDX6 siRNA, there was no apparent increase of cytotoxicity when compared to the control siRNA. To account for the non-specific cytotoxic effects, results of viral infectivity and biochemical analyses were always normalized to the levels of GAPDH and Gag in cells. As shown in Fig. 1A, 90-98% of protein expression was reduced for each gene when cells were transfected with 120 nM siRNA. Knockdown of DDX6 had a dramatic effect on viral infectivity with a 33-fold reduction while depletion of PABPC1 caused a 5.5-fold decrease in titer (Fig. 1B). In contrast, infectivity was increased by 5.6 fold when Dcp1 was silenced. Knockdown of MOV10, Ago1, or Ago2 had no effect on virus titers. The effect of DDX6 knockdown on viral infectivity was similar no matter whether cells were infected with PFV or transfected with pcPFV, an infectious clone (data not shown). Since transfection with a proviral DNA bypasses the early events in the infectious cycle such as adsorption, penetration, uncoating, or integration, the effects of DDX6 knockdown are unlikely caused by interruption of an early step in infection. Thus, some, but not all, P body and stress granule proteins are critical for the replication of foamy virus.

### DDX6 specifically re-localizes to the viral assembly site

**Figure 2:**
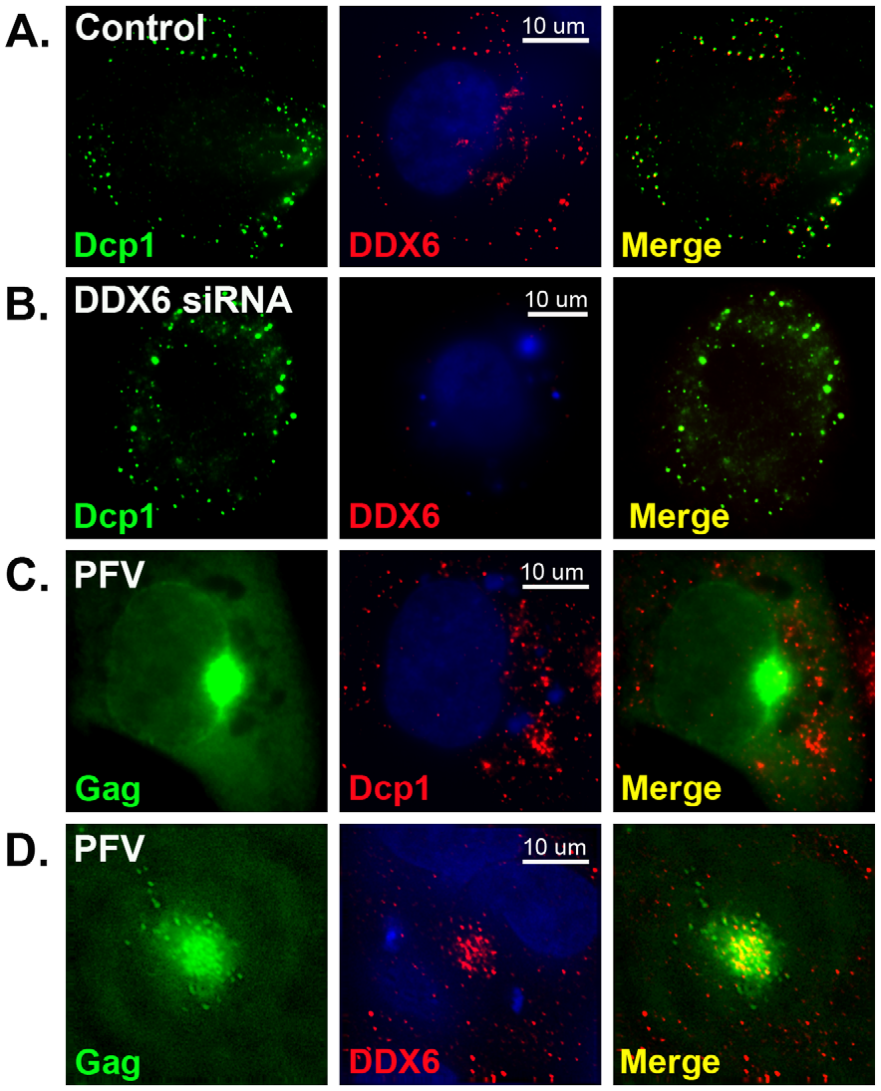
Immunofluorescent staining of DDX6 and Dcp1. HT1080 cells were mock-transfected (A), DDX6 siRNA-transfected (B), or transfected with pcPFV/gag-gfp (C & D) for 32 hrs, and stained with anti-Dcp1 (A, B, & C) or anti-DDX6 (A, B, & D). Gag-GFP proteins were imaged as green fluorescent color (Left panels, C & D). After merged together, the yellow color indicates co-localization of green and red signals. Images were taken from a single 0.2 um Z- section using DeltaVision microscopy.

For yeast retrotransposons Ty1 and Ty3, their viral RNAs, Gag proteins, and virus-like particles all accumulate in P bodies where particle assembly and RNA packaging occur [13]–[15]. We asked if there was any association of cytoplasmic P bodies and stress granules or their components with foamy virus assembly site. HT1080 cells were infected with PFV or transfected with proviral DNA pcPFV/gag-gfp where the GFP polypeptide was inserted at the C-terminus of Gag. Cells were then fixed and stained with specific primary antibodies to detect endogenous cellular proteins. In cells, DDX6 is often concentrated in both P bodies and stress granules where Dcp1 is a commonly used marker to detect P bodies. In the absence of foamy virus infection, DDX6 typically co-localized with Dcp1 in cytoplasmic P body granules (Fig. 2A). Transfection with DDX6 siRNA caused little change in distribution of Dcp1-associated P-bodies even though expression of endogenous DDX6 was clearly suppressed (Fig. 2B). When cells were infected with PFV or transfected with proviral DNA pcPFV/gag-gfp, Gag proteins were accumulated near the MTOC (Fig. 2C & 2D) where particles were assembled as previously reported [4], but there was no co-localization of Gag with Dcp1 or Dcp1-associated P body granules (Fig. 2C). Similarly, we found no difference in distribution of other P body and stress granule proteins such as Dcp2, GW182, Ago2, and PABPC1 after PFV infection (data not shown). However, the localization of DDX6 dramatically changed in the presence of foamy virus Gag in that an intense staining of DDX6 was detected at the MTOC area where Gag capsid proteins were concentrated (Fig. 2D). We quantified the amount of DDX6 that re-localized to the assembly site after viral infection or transfection (see Materials and Methods), and found that about 15–25% of total endogenous DDX6 proteins were located in the MTOC area after infection. Therefore, specific re-localization of DDX6, but not the other P-body or stress granule proteins tested, to the viral capsid assembly site implies a possible role of DDX6 in the life cycle of foamy virus.

**Figure 3:**
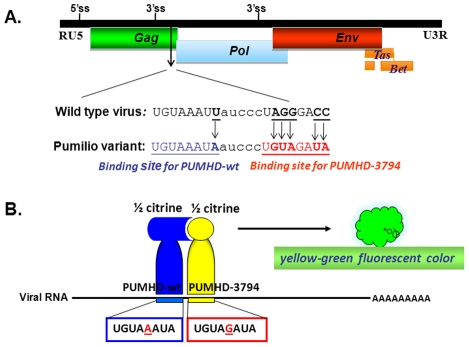
Schematic presentation of the pumilo-based BIFC system designed for pcPFV/gag-pum. (A) Two eight-nucleotide sequences (TGTAAATA & TGTAGATA) that were separated by 5 nucleotides were introduced at the 3′ end of *gag* in the proviral DNA pcPFV/gag-pum. Consequently, all viral genomic RNAs as well as unspliced *gag* and singly spliced *pol* mRNAs contained UGUAAAUA and UGUAGAUA in their sequences. (B) These motifs were target substrates for wild-type PUMHD(wt) and a variant PUMHD(3794) that had been fused separately to either the C- or N-terminal half of mCitrine, a yellow-green fluorescent protein. When pcHFV/gag-pum was co-transfected with expression vectors pcmv-PUMHD(wt)_CitC and pcmv- CitN_PUMHD(3794), viral RNA containing PUMHD-binding sequences is viewed in green fluorescent color.

**Figure 4:**
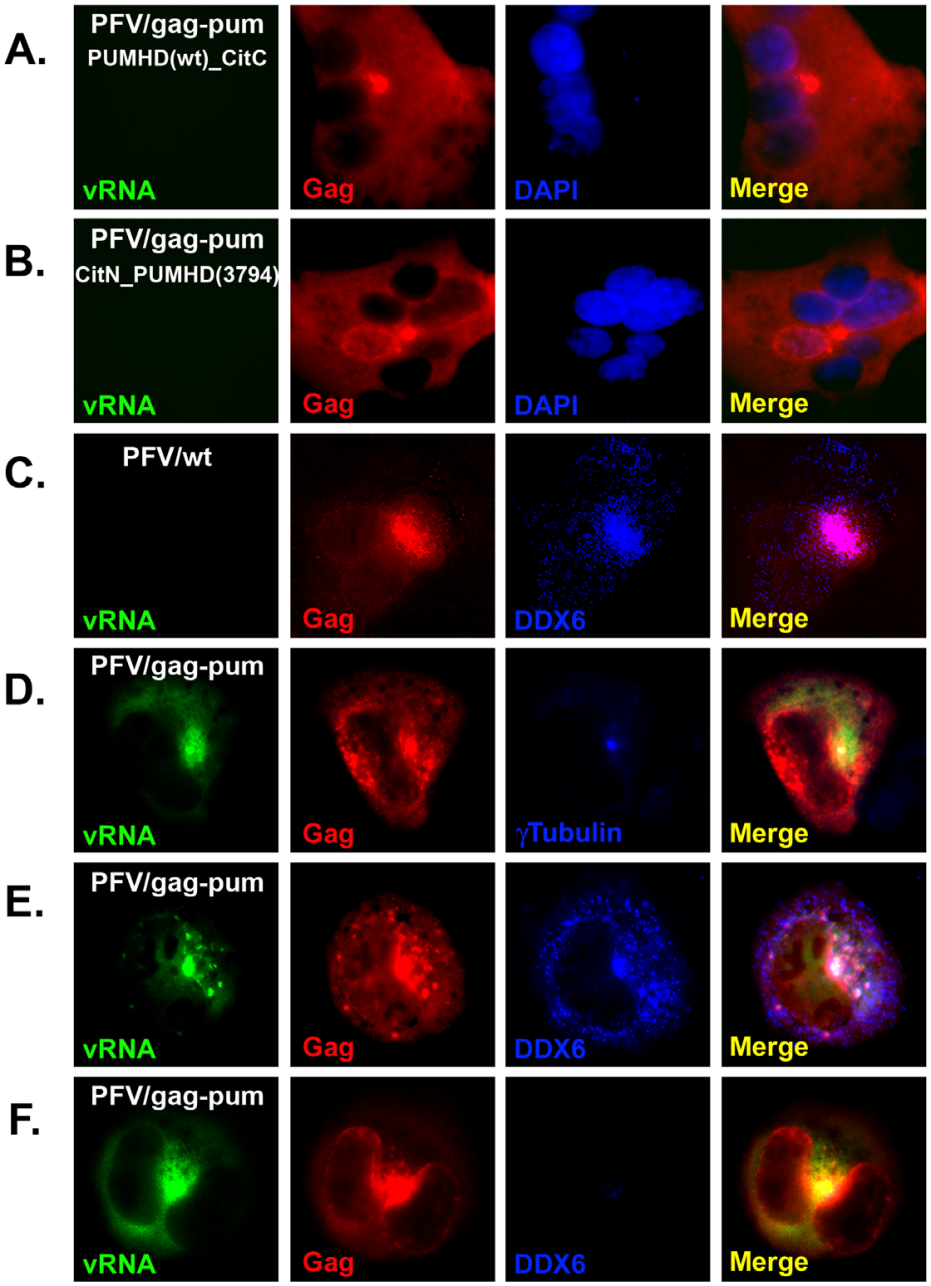
Co-localization of DDX6 with viral RNA and Gag near the MTOC. HT1080 was co-transfected with pcPFV/gag-pum plus either pcmv-PUMHD(wt)_CitC (A) or pcmv-CitN_PUMHD(3794) (B), or co-transfected with pcPFV-wt (C) or pcPFV/gag-pum (D, E, & F) plus both expression vectors pcmv-PUMHD(wt)_CitC and pcmv-CitN_PUMHD(3794). At 32 h after transfection, cells were stained with rabbit anti-Gag (A to F) and mouse anti-DDX6 (C, E, & F) or mouse anti-γ-tubulin (D). Cells in Panel F were first treated with DDX6 siRNA before co-transfected with pcPFV/gag-pum and both expression vectors. Viral RNA containing PUMHD-binding sequences was viewed in green color (Left panel, D to F). Images were captured using DeltaVision microscopy.

DDX6 is a RNA binding protein and a RNA helicase, leading to a hypothesis that DDX6 is involved in transporting viral elements, particularly viral RNA, to the viral assembly site. To characterize subcellular localization of foamy virus RNA *in vivo*, we adapted a pumilio-based bimolecular fluorescence complementation (BiFC) reporter system developed by Tilsner *et al.* [26]. Pumilio is a sequence-specific RNA binding protein. The RNA-binding domain of pumilio, PUMHD, consists of eight three-amino-acid repeats that recognize 8-base RNA sequence UGUANAUA. Because each three-amino-acid repeat binds to a single RNA base, the substrate specificity of PUMHD can be altered by changing amino acid residues within each repeat. In order to increase binding specificity and reduce background signals, wild type and a mutant PUMHD are fused separately to split halves of a fluorescent protein. Only when both polypeptides concurrently bind to the two neighboring recognition motifs in the RNA it can bring the split halves of the fluorescent protein into a close proximity to generate a specific fluorescent signal. For PFV, two adjacent 8-bp sequences, TGTAAATA and TGTAGATA, were introduced at the 3′ end of *gag*, resulting in minimal changes from the wild-type sequences. Consequently, all viral genomic RNAs as well as unspliced *gag* and singly spliced *pol* mRNAs contained UGUAAAUA and UGUAGAUA in their sequences (Fig. 3A). These motifs were target substrates for wild-type PUMHD(wt) and a variant PUMHD(3794) that had been fused separately to either the C- or N-terminal half of mCitrine, a yellow-green fluorescent protein. Binding of both PUMHD polypeptides to target sequences in the viral RNA could lead to the BiFC effects and thus allow detection of virus-specific RNA (Fig. 3B). Virus derived from this modified DNA pcPFV/gag-pum was as infectious as wild type pcPFV. No background signal was found when pcPFV/gag-pum was co-transfected with only one expression vector (either PUMHD-wt or PUMHD-3794) (Fig. 4A & 4B). The background was also very low when wild type pcPFV was co-transfected with both expression vectors pcmv-PUMHD(wt)_CitC and pcmv- CitN_PUMHD(3794) (Fig. 4C). In contrast, co-transfection of pcPFV/gag-pum with both expression vectors produced fluorescent signals that are lightly dispersed throughout the cytoplasm (Fig. 4D, 4E, & 4F), reflective of ribosome-bound *gag* and *pol* mRNA. Noticeably, a higher concentration of viral RNA was detected at the MTOC area (as stained by γ-tubulin antibody) (Fig. 4D) where Gag and DDX6 were co-localized (Fig. 4E). Interestingly, DDX6 knockdown had little effect on the concentration of viral RNA and Gag near the MTOC (Fig. 4F). These results show that DDX6 co-localizes with viral RNA and Gag at the viral assembly site but DDX6 is not required for trafficking of viral RNA or Gag to the MTOC area.

### DDX6 knockdown affects assembly of viral RNA into extracellular particles

**Figure 5:**
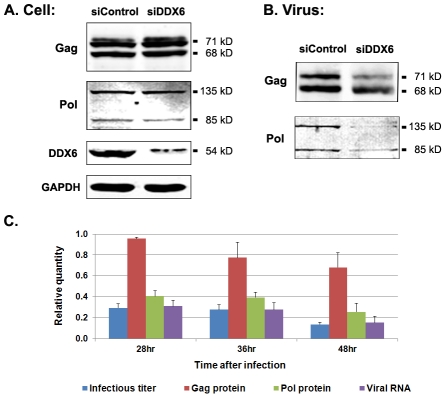
Viral proteins and RNA in cell lysates and extracellular particles after DDX6 knockdown. HT1080 cells were transfected twice with 60 nM control siRNA or DDX6 siRNA. 1^st^ siRNA transfection was done at time 0. 2^nd^ siRNA transfection was performed at 24 h, followed by the infection with foamy virus at 48 h (i.e. 24 h after 2^nd^ siRNA transfection). Cells and culture supernatants were harvested at 45 h after infection. (A) Immunoblot analyses of Gag, Pol, DDX6, and GAPDH in infected cell lysates. (B) Immunoblot analyses of Gag and Pol in extracellular particles. (C) Relative quantities for viral infectivity, Gag and Pol proteins, and viral RNA in extracellular particles obtained from transfection with DDX6 siRNA compared to that with control siRNA. Means and standard errors were results for each analysis from at least four independent experiments.

To identify the role of DDX6 in viral replication, we examined viral RNA and proteins in both intracellular and extracellular fractions from DDX6-knockdown cells. When cells were transfected twice with 120 nM siRNA as shown in Figure 1, more than 98% of DDX6 was depleted and viral infectivity was reduced for more than 30 fold. To minimize cytotoxicity from the siRNA for biochemical analyses, 60 nM siRNA was used and about 85% of DDX6 was reduced (Fig. 5A) and virus titers were decreased 3–8 fold (Fig. 5C). Under this condition, the levels of Gag and Pol in the cell lysates were minimally affected as shown in Fig. 5A where the lower band of Gag, p68, represented the cleavage product of Gag precursor protein, p71 (the upper band). Culture supernatants from cells that were treated with 60 nM control or DDX6 siRNA and subsequently infected with PFV were filtered and pelleted through 20% sucrose cushion by ultracentrifugation. The amounts of supernatants that were used to pellet the viruses were adjusted for each sample according to the level of intracellular Gag proteins that had been normalized to the level of the cellular protein GAPDH. Despite an 8-fold reduction in viral titer, the pelleted virus particles contained similar amounts of Gag with or without DDX6 (Fig. 5B), indicating that viral protein expression and particle assembly and release were relatively unaffected by DDX6 siRNA. However, the level of Pol in these extracellular particles was decreased even though proteolytic cleavage of p71 Gag into p68 was efficient (Fig. 5B). Since it has been shown that foamy virus protease can be activated in the absence of Pol packaging into assembling particles [27], proteolytic cleavage of Gag precursor p71 into p68 could occur in cells even without assembly of Pol into virions. When equal numbers of virus particles from each sample (determined by its extracellular Gag level) were analyzed by quantitative RT-PCR, the level of viral nucleic acids in the pelleted extracellular particles was much lower after DDX6 knockdown (Fig. 5C). It is worth noting that different from other retroviruses, assembly of foamy virus Pol into capsids requires viral genomic RNA [28]. A decrease in Pol packaging after DDX6 knockdown (Fig. 5B) was thus consistent with a lower level of virus genome assembled into virions. These data suggest that DDX6 plays an important role in viral RNA packaging.

### DDX6 does not interact stably with Gag nor is it incorporated into particles

**Figure 6:**
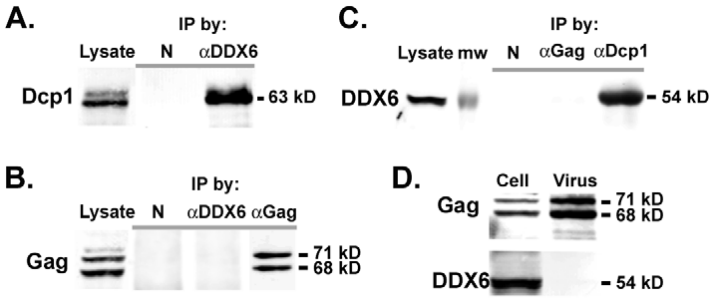
Co-immunoprecipitation of DDX6 with Dcp1 but not with Gag. (A) 293T cells were transfected with pcPFV for 45 hrs. Cleared cell lysates were immunoprecipitated with normal rabbit serum (referred to as N) or rabbit anti-rck/p54 (referred to as αDDX6), and analyzed by immunoblot using mouse anti-Dcp1. (B) Immunoprecipitation of transfected cell lysates using normal rabbit serum (N), rabbit anti-rck/p54 (αDDX6), or rabbit anti-Gag, and analyzed by immunoblot with mouse anti-Gag. (C) Cell lysates were immunoprecipitated by normal rabbit serum (N), rabbit anti-Gag, or rabbit anti-hDcp1a (referred to as αDcp1), and analyzed by immunoblot with mouse anti-DDX6 antibody. (D) HT1080 cells were infected with virus at an moi of 2 for 45 hrs and subjected to immunoblot analyses using rabbit anti-Gag and mouse anti-DDX6 in the infected cell lysates and extracellular virus particles.

To understand the specific role of DDX6 in the assembly of virus genome, we wanted to find out whether DDX6 interacted with the viral RNA or Gag capsid proteins. Although DDX6 co-localized with Gag at the assembly site, it was unclear if DDX6 bound to Gag proteins directly. Cell lysates from pcPFV-transfected 293T cells were immunoprecipitated using anti-DDX6 or anti-Gag antibody. Because Dcp1 was known to physically interact with DDX6 [29], [30], it was used as a positive control for co-immunoprecipitation with DDX6. Our results show that DDX6 was readily immunoprecipitated with Dcp1 (Fig. 6A & 6C), but not with Gag (Fig. 6B, & 6C), indicating that there was no stable interaction of DDX6 with Gag capsid proteins. We then asked whether DDX6, known to be a RNA binding protein, binds to the viral RNA. However, there was very little evidence of selective binding of DDX6 to any particular mRNA sequences or species [16], [19]. It was thus difficult to discern binding of DDX6 to the viral RNA from other cellular RNA substrates. Nonetheless, whether the binding of DDX6 to the viral RNA was sequence specific or not, one possible consequence was that DDX6 was incorporated into particles through a stable interaction with the viral genomic RNA. Surprisingly, although DDX6 was abundant in infected cell lysates, it was below the detection level in pelleted extracellular particles (Fig. 6D), indicating that binding of DDX6 to viral RNA occurred either transiently or at a very low copy number.

### The ATPase/helicase activity of DDX6 is required for viral replication

**Figure 7:**
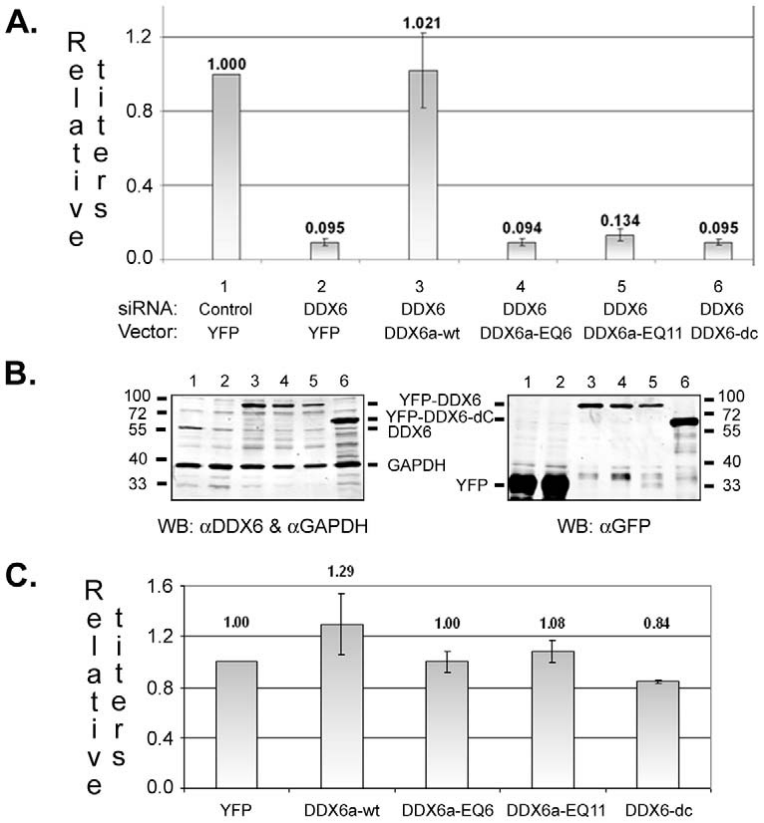
Exogenous expression of wild type or mutant DDX6. (A & B) HT1080 cells were transfected with control siRNA (Lanes 1) or DDX6 siRNA (Lanes 2 to 6) for 24 hrs. Cells were then transfected with pEYFP (Lanes 1 & 2), pEYFP-DDX6a-wt (Lanes 3), pEYFP-DDX6a-EQ6 (Lanes 4), pEYFP-DDX6a-EQ11 (Lanes 5), or pEYFP-DDX6-dC (Lanes 6). At 24 hr later, cells were infected with the virus at an moi of 2 for 6 hrs, washed, and incubated for another 40 hrs. Culture supernatants were assayed by FAB indicators cells to measure the virus titers. (A) Viral infectivity relative to the transfection with control siRNA and pEYFP (Lane 1). (B) Immunoblot analyses of GAPDH, endogenous DDX6, and vector-expressed YFP or YFP-DDX6 proteins in infected cell lysates. Molecular weight standards are marked for each gel. (C) HT1080 cells were transfected with pEYFP, pEYFP-DDX6a-wt, pEYFP-DDX6a-EQ6, pEYFP-DDX6a-EQ11, or pEYFP-DDX6-dC, and then infected with the virus at an moi of 2. Results are shown as relative viral infectivity compared to the transfection with the control vector pEYFP. Means and standard errors were results for each analysis from at least four independent experiments.

*In vitro*, DDX6 has RNA-dependent ATPase and ATP-dependent RNA helicase activities that are determined by the highly conserved DEAD box as well as sequences in its C-terminal domain [17]. To test if the ATPase/helicase activity of DDX6 was required for viral replication, wild type or mutant DDX6 was expressed from a vector after endogenous DDX6 was silenced by the siRNA. Both DDX6-EQ (a point mutation in the DEAD box) and DDX6-dC (183 aa deletion at the C-terminus) lack ATPase and helicase activities [19]. To prevent binding of siRNA to DDX6's sequences in the vectors, siRNA binding targets in wild type pEYFP-DDX6a-wt and two separate clones of EQ mutants (pEYFP-DDX6a-EQ6 and EQ11) were mutated yet maintained wild type amino acid sequences. The siRNA binding site is absent in pEYFP-DDX6-dC since the sequences reside in the region that is deleted in the mutant. Expression of wild type DDX6 from the vector pEYFP-DDX6a-wt successfully rescued viral infectivity after siRNA knockdown of endogenous DDX6 (Fig. 7A, Lanes 1–3). However, none of the helicase mutants DDX6a-EQ6, DDX6a-EQ11, and DDX6-dC could restore virus titers (Fig. 7A, Lanes 4–6) even though their expression levels were similar to DDX6a-wt (Fig. 7B, Lanes 3-6). In contrast, exogenous expression of wild-type DDX6 in the presence of endogenous DDX6 (without siRNA transfection) led to only a modest increase in virus titers and the helicase mutants had little effects (Fig. 7C), indicating that DDX6 is not limiting for viral infection and the helicase mutants did not have dominant negative effects. Thus, these results indicate that the intrinsic ATPase/helicase activity of DDX6 is essential for the replication of prototype foamy virus.

## Discussion

In this study, we find that the RNA helicase DDX6, a cellular protein associated with P bodies as well as stress granules, is re-directed to the site of foamy virus assembly and plays an important role in assembly of viral RNA into particles. An intact ATPase/helicase domain of DDX6 is essential for viral replication, suggesting that the ATP hydrolysis and RNA unwinding activities of DDX6 function in rearranging the structure of viral RNA and/or RNA-Gag complex into a proper conformation to facilitate viral RNA packaging.

About 20% of endogenous DDX6 proteins, but not other P body/stress granule proteins tested, are re-located to the viral assembly site near the MTOC where DDX6 co-localizes with viral RNA and Gag capsid proteins. We have ruled out a possible role of DDX6 in transporting viral RNA and Gag to the MTOC area, and it is unclear how DDX6 is re-localized to the assembly site which occurs specifically only after virus infection or transfection. DDX6 could bind to Gag protein and/or viral RNA and be transited to the MTOC area. However, we have no evidence for a direct, stable interaction between DDX6 and Gag (Fig. 6). The observations that DDX6 does not bind to Gag stably and its RNA-dependent ATPase/helicase activity is essential for viral replication strongly imply that DDX6 interacts with viral RNA for its function in RNA packaging. It is possible that a specific ribonucleoprotein complex is formed through binding of DDX6 to the viral RNA, leading to segregation of the genomic RNA for packaging from mRNA translation. It is also possible that binding of DDX6 occurs via *cis*-acting packaging sequences in the viral RNA to promote its recognition by Gag. More studies will be needed to clarify these interactions and to understand the exact role of DDX6 in the process of viral RNA encapsidation.

The biological functions of DDX6 *in vivo* are complex. Although generally known as a translational repressor [31], [32], DDX6 can interact with other factors including Dcp1 to increase efficiency of the decapping enzyme Dcp2 [33], and is also implicated as a proto-oncogene [34] as well as a regulator at the G1/S transition [35]. Although DDX6 is known as a non-specific RNA binding protein, selective binding of DDX6 could occur through interactions with other factors bound to the same RNA substrate [36]. The specific functions of DDX6 seem to be critically influenced by its binding partners as well as the cellular environments. It is believed that the diversity of DDX6's functions *in vivo* is due to its ability to mediate the conformational change of its RNA substrates and thus affect the fate of DDX6-complexed RNA [16]. Therefore, it is very possible that the role of DDX6 in viral RNA packaging involves other protein partners, cellular or even viral proteins. Even though we have no evidence of a direct, stable interaction between DDX6 and Gag by co-immunoprecipitation (Fig. 6), we cannot rule out the possibility that binding of DDX6 to Gag occurs transiently. Further experiments such as CLIP (*in vivo* cross-linking and immunoprecipitation) could help in addressing this question.

We have also identified two other cellular proteins that affect foamy virus replication; silencing of the poly(A)-binding protein PABPC1 (a stress granule protein) results in a decrease in viral infectivity. PABPC1 is a translation initiation factor but is also involved in many aspects of mRNA metabolism [37]. In contrast, knockdown of Dcp1 (a P body protein) leads to an increase in virus titers, suggesting that Dcp1 is a negative factor for viral replication. It is possible that Dcp1 acts to negatively regulate viral replication by sequestering DDX6 in a protein-protein complex. Alternatively, Dcp1, an activator of the 5′-3′ mRNA decapping enzyme Dcp2, could enhance decapping and subsequent destabilization of viral genomic RNA. More studies are needed to understand the specific role of Dcp1 on foamy virus replication. In summary, the results in this study have revealed a unique and dynamic interaction between P body/stress granule proteins and foamy virus infection where Dcp1 negatively regulates viral RNA packaging but DDX6 is required for efficient encapsidation of virus genomes.

## Materials and Methods

### Cells and viruses

HT1080, 293T, and FAB cells were grown in Dulbecco's modified Eagle's media (DME) supplemented with 10% fetal bovine serum. All virus stocks were derived from an infectious clone of prototype foamy virus, pcPFV [38]. Virus titers were determined using FAB indicator cells [25].

### Antibodies

Rabbit polyclonal anti-Gag and mouse monoclonal anti-Pol have been described previously [4]. Rabbit polyclonal anti-hDcp1a was a gift from Jens Lykke-Andersen (U. Colorado, CO). Rabbit polyclonal anti-rck/p54 was from MBL (Woburn, MA). Mouse monoclonal anti-DDX6 and anti-Dcp1 were from Abnova (Taipei, Taiwan). Mouse monoclonal anti-PABPC1 and anti-GAPDH were from Santa Cruz (Santa Cruz, CA). Mouse monoclonal anti-Dcp2, anti-Ago2, anti-GW182, and anti-γ-tubulin were from Abcam (Cambridge, MA). Goat anti-mouse or anti-rabbit secondary antibodies conjugated with Alexa Fluor 488, 555, and 680 were from Invitrogen (Carlsbad, CA).

### Plasmids

Proviral construct pcPFV/gag-gfp was generated by inserting a copy of the *gfp* gene at the 3′ end of *gag*, creating a polypeptide with GFP fused at the 631^st^ amino acid of Gag. To generate pcPFV/gag-pum, an oligonucleotide pair of 5′-GACCATCAGATCAAGGTTCTGTAAATACTAGGCCTGTAGATAATCAAGCAGGCTCTGGG-3′ and 5′-CCCAGAGCCTGCTTGATTATCTACAGGCCTAGTATTTACAGAACCTTGATCTGATGGTC-3′ was used to create two adjacent eight-nucleotide sequences (TGTAAATA and TGTAGATA) at the 3′ end of *gag*. To create BiFC effects, the sequences encoding the split Citrine halves that had been separately fused to either the wild-type or mutant PUMHD domain were cloned into a mammalian expression vector. Thus, pcmv-PUMHD(wt)_CitC and pcmv- CitN_PUMHD(3794) were made by inserting an AflII/XhoI fragment from pGEMTE.PUMHD_CitC or pGEMTE.CitN_PUMHD3794 (gifts from J Tilsner and K Oparka, U. Edinburg, UK) into an AflII/XhoI digested pcDNA3.1 vector.

### siRNA

siRNAs specific to DDX6, Dcp1, PABPC1, Ago1, Ago2, and MOV10, as well as control siRNAs (siCONTROL Non-Targeting siRNA #1 and #5) were from Dharmacon (Lafayette, Colorado), and some were supplied by Dr. Vineet KewalRamani (NCI, Frederick) . DDX6: 5′-CGAAAUGGCUUAUGCCGCAUU-3′ or 5′-GCAGAAACCCUAUGAGAUU-3′; Dcp1a: pool of 5′-GCAAGCUUGUCGAUAUAUAUU3′, 5′-ACUCAUGGCUGAUGUGGUAUU-3′, 5′-ACAAGCAUCUGACGGUAGAUU-3′, and 5′-CCAAUUCAUUCCUACCAUUUU-3′; PABPC1: pool of 5′-CAUGUAAGGUGGUUUGUGAUU-3′, 5′-GAGCAAGGAAACGUAAUUUUU-3′, 5′-GGACAAAUCCAUUGAUAAUUU-3′, and 5′-UGGAUGAGAUGAACGGAAAUU-3′; MOV10: pool of 5′-CGGCAAGACUGUCACGUUAUU-3′, 5′-GGUCAGAUAUCAGCAAACAUU-3′, 5′-GCCAUGAGGCACAUUGUUAUU-3′, and 5′-CAAUUAAGCAGGUGGUGAAUU-3′; Ago1: pool of 5′-GCACAGUAUUUCAAGCAGAUU-3′, 5′-CAACGAACGGGUCGACUUUUU-3′, 5′-UGACAAGAAUGAGCGAAUUUU-3′, and 5′-GGAAGUACCGCGUGUGUAAUU-3′; Ago2: pool of 5′-GCACGACUGUGGACACGAAUU-3′, 5′-CCAAGGCGGUCCAGGUUCAUU-3′, 5′-GGUCUAAAGGUGGAGAUAAUU-3′, and 5′-CAAGCAGGCCUUCGCACUAUU-3′.

### siRNA Transfection

HT1080 cells were seeded at 1×10^5^ cells per well in a 6-well plate. The next day, 4 ul of Lipofectamine RNAiMAX (Invitrogen, Carlsbad, CA) was mixed with 11 ul of sera-free, antibiotics-free DME and incubated for 10 min at RT. This mixture was then combined with 10 ul of the siRNA (either 60 or 120 uM) that had been diluted in 175 ul of sera-free, antibiotics-free DME. After 20 min at RT, the mixture was added to the cells in 0.8 ml of sera-free, antibiotics-free DME. After incubation for 6 hrs at 37°, the complex was removed and replaced with 3 ml of complete media. Cells were transfected with the siRNA again the next day to maximize the knockdown effects.

### Infection and immunoblot

24 hours after the second siRNA transfection, cells were infected with virus at an moi of 2 for 6 hrs, washed, and replaced with fresh media. Virus titers in culture supernatants were determined by the FAB assay [25]. Half of the cells were subjected to quantitative Western blot analysis using the Odyssey detection system (Li-Cor, Lincoln, NE) according to the manufacturer's protocol. The other half of the sample was used to extract RNA that was analyzed by quantitative real-time RT/PCR. Intracellular protein and RNA levels were normalized to the level of GAPDH protein or RNA respectively as internal controls.

### Immunofluorescence

5×10^4^ HT1080 cells seeded in a 12-well plate were infected with virus, transfected with 0.7 ug pcPFV or pcPFVgag-gfp, or co-transfected with 0.5 ug pcPFV/gag-pum, 0.1 ug pcmv-PUMHD(wt)_CitC and pcmv- CitN_PUMHD(3794) using Polyfect (QIAGEN, Valencia, CA). Cells were fixed and stained by primary antibodies and Alexa Fluor-conjugated secondary antibodies as described previously [4]. Fluorescent images were first visualized using a fixed-stage Nikon Eclipse E800 microscope. Three dimensional images in Z-stacks (a series of images with the same X and Y coordinates but varying along the vertical focus or Z axis) are captured and reconstructed using Deltavision RT wide-field deconvolution microscopy, which is equivalent to confocal microscopy. Quantitative analysis of co-localization was performed using the full set of 3-D data obtained from Deltavision microscopy by Volocity Quantization software (PerkinElmer, Waltham, MA).

### Extracellular virus particles

Culture supernatants were filtered and pelleted through 20% sucrose cushion by ultracentrifugation to obtain extracellular particles. The amounts of supernatants to be pelleted were determined according to each sample's intracellular Gag level that was first normalized by GAPDH. The pelleted material was treated with 0.5 mg/ml substilisin (Sigma, St Louis, MO) at 37°C for 2 hrs to remove non-specific aggregates, mixed with 0.5 mg/ml PMSF to stop the reaction, and analyzed by Odyssey Western blot.

### Quantitative RT/PCR

Total cellular RNAs were extracted using RNeasy Mini Kit (QIAGEN). To obtain RNA from the extracellular particles, equal numbers of particles in each sample, as determined by its extracellular Gag level, were treated with RNase-free DNaseI before using QIAamp Viral RNA Mini Kit (QIAGEN). Viral RNA or 1 ug of total cellular RNA from each sample was reverse transcribed using random primers and ThermoScript RT-PCR system (Invitrogen), and subjected to quantitative PCR reactions in triplicates using SYBR Green PCR Core Reagents and ABI 7900HT Real Time PCR Systems (Applied Biosystems, Foster City, CA). In the quantitative PCR reactions, GAPDH was used as an internal control for cellular RNA with a primer set of 5′-CTACTGGCGCTGCCAAGGCTGT-3′ and 5′-GCCATGAGGTCCACCACCCTGT-3′. Primer sets of 5′-CATAGCGGGACCCGTATAAAAG-3′ and 5′-CAACCAGAGCTTCAACATCAAG-3′ were used to detect unspliced *gag* RNA.

### Immunoprecipitation

293T cells were transfected with pcPFV for 45 hrs. Cells were washed by ice-cold PBS and incubated for 30 min on ice in a lysis buffer containing 100 mM Tris, pH 7.4, 100 mM NaCl, 10 mM EDTA, 50 mM KAc, 0.625% of NP40, and protein inhibitors. Lysates were centrifuged at 2000 rpm for 10 min followed by another centrifugation at 12,000 rpm for 2 min. The cleared lysates were then incubated with normal rabbit serum, rabbit anti-rck/p54 (referred to as anti-DDX6), anti-hDcp1a, or anti-Gag at 4° for 2 hrs before addition of protein A sepharose (GE Healthcare, Piscataway, NJ). After incubation overnight, the beads were washed 5 times with a wash buffer containing 100 mM Tris, pH 8.0, 100 mM NaCl, and 10 mM EDTA, eluted in 2× SDS-PAGE sample buffer, and analyzed by Immunoblot using mouse anti-Dcp1, mouse anti-DDX6, or mouse anti-Gag.

### Exogenous expression of DDX6

Expression vector pEYFP-RCK/p54 (referred to as pEYFP-DDX6-wt) was a gift from T. Rana (U. Massachusetts, Worcester, MA). pEYFP-DDX6-EQ and pEYFP-DDX6-ΔC (referred to as pEYFP-DDX6-dC) were gifts from S. Lemon (U. Texas, Galveston, TX). pEYFP-DDX6a-wt and pEYFP-DDX6a-EQ were created using primers (5′-TCGTGTATTTCATGATTTCCGGAACGGTCTCTGTAGCAATCTTGTTTGCACTGATC-3′ and 5′- GATCAGTGCAAACAAGATTGCTACAGAGACCGTTCCGGAAATCATGAAATACACGA-3′) that contains 7 changes in the binding sequences to DDX6 siRNA yet maintains wild type amino acid sequences. 5×10^4^ HT1080 cells per well of a 12-well plate were treated with 120 nM DDX6 siRNA for 24 hrs, transfected with 0.6 ug pcEYFP-C1, or 0.8 ug pEYFP-DDX6a-wt, pEYFP-DDX6a-EQ, or pEYFP-DDX6-dC for 24 hrs, infected with virus at an moi of 2 for 6 hrs, washed, and incubated for another 40 hrs.
